# Recurrent Acute Calculous Cholecystitis in a Retained Gallbladder Remnant: A Case Report

**DOI:** 10.7759/cureus.104508

**Published:** 2026-03-01

**Authors:** Breanna Nowak, Kristin J Brown

**Affiliations:** 1 Emergency Medicine, Ascension Saint Thomas Rutherford, Murfreesboro, USA

**Keywords:** abdominal pain, acute cholecystitis, case report, gallbladder remnant, subtotal cholecystectomy

## Abstract

We present the case of a 67-year-old woman with a history of prior laparoscopic cholecystectomy who presented with right upper quadrant (RUQ) pain and nausea and was found to have recurrent acute cholecystitis. Review of the operative note from her previous cholecystectomy revealed that a rim of gallbladder tissue had been left behind due to anatomical complications. CT of the abdomen and pelvis demonstrated a distended gallbladder with cholelithiasis, wall thickening, and pericholecystic fluid, consistent with acute calculous cholecystitis despite her history of prior cholecystectomy. The patient was admitted and later underwent definitive laparoscopic total cholecystectomy. Intraoperatively, extensive RUQ adhesions were taken down, the cystic duct was identified and clipped, and the gallbladder remnant was excised. This case highlights the importance of considering gallbladder remnant acute cholecystitis in patients with prior subtotal cholecystectomy who present with RUQ pain.

## Introduction

Incomplete cholecystectomy leading to a retained gallbladder remnant is an uncommon but important cause of recurrent biliary pathology, including cholecystitis and biliary colic. The rates of laparoscopic cholecystectomy have increased, which in turn has led to a higher incidence of subtotal cholecystectomy (STC). This procedure is commonly performed in the presence of inflammation, abnormal anatomy, or adhesions to reduce the risk of bile duct injury [1]. While STC allows for safer gallbladder removal, the retained tissue predisposes patients to subsequent biliary pathology, including biliary colic, recurrent cholecystitis, choledocholithiasis, and pancreatitis [2,3].

Two types of STC are utilized: fenestrating and reconstituting. Fenestrating STC may involve suturing of the cystic duct while leaving the gallbladder open to drain, whereas the reconstituting technique closes the gallbladder with sutures or staples. The reconstituting approach reduces the risk of bile leakage and biliary fistulas but increases the risk of recurrent gallstone formation and subsequent biliary disease, as demonstrated in the present case [4].

In the ED, patients with prior cholecystectomy are often presumed to have no gallbladder pathology; however, residual gallbladder tissue may still harbor stones and become inflamed [3,5]. This scenario presents unique diagnostic and management challenges. CT and ultrasound findings may be misinterpreted in the setting of a gallbladder remnant, and magnetic resonance cholangiopancreatography (MRCP) is particularly useful for identifying underlying pathology [3,6].

We present a case of acute calculous cholecystitis of a gallbladder remnant in a 67-year-old woman with a history of STC. This case highlights the importance of maintaining a high index of suspicion for gallbladder remnant pathology and discusses implications for ED management.

## Case presentation

A 67-year-old woman with a past medical history of hypothyroidism and prior appendectomy and cholecystectomy presented with right upper quadrant (RUQ) abdominal pain and nausea of approximately 12-hour duration. The pain was described as a dull ache, and she reported no vomiting.

Eight years prior (2017), the patient had undergone a laparoscopic reconstituting STC, during which dissection planes could not clearly identify the cystic duct, and a rim of gallbladder tissue was left behind.

On presentation in July 2025, her vital signs were temperature 98.3 °F (36.8 °C), heart rate 66 beats per minute, respiratory rate 16 breaths per minute, blood pressure 154/96 mmHg, and SpO₂ 98% on room air. Physical examination revealed minimal RUQ tenderness without rebound or guarding. Laboratory evaluation showed a slightly elevated white blood cell count, while liver function tests, lipase, and bilirubin were within normal limits (Table 1).

**Table 1:** Laboratory results on initial evaluation

Parameter	Value	Reference range
White blood cell	13.4 × 10³/mm³	4.5-10.3 × 10³/mm³
Hemoglobin	14.1 g/dL	12.0-16.0 g/dL
Total bilirubin	0.7 mg/dL	0.2-1.2 mg/dL
Alkaline phosphatase	102 U/L	40-150 U/L
Aspartate aminotransferase	16 U/L	5-34 U/L
Alanine aminotransferase	12 U/L	≤55 U/L
Lipase	20 U/L	8-78 U/L

**Figure 1:**
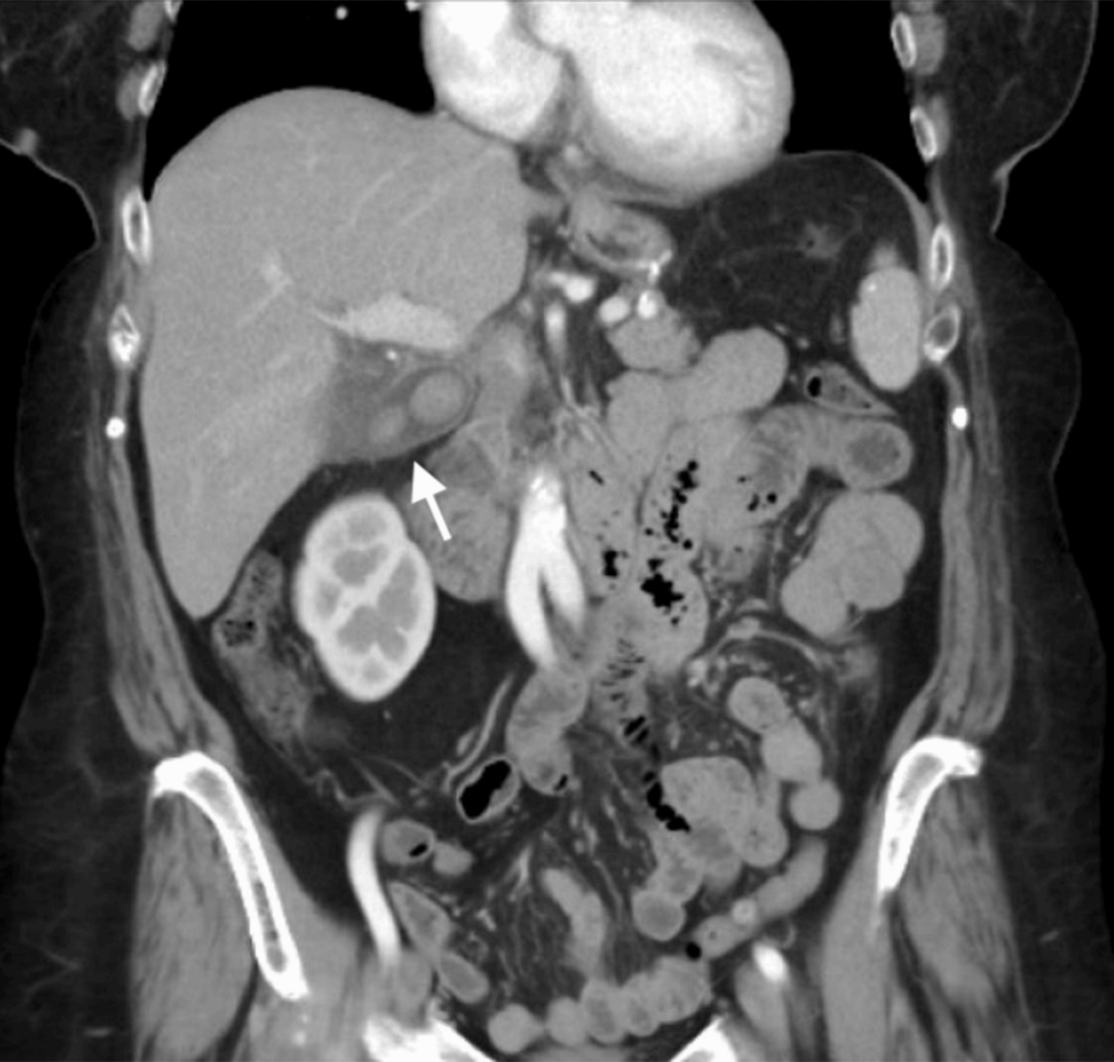
Coronal CT image showing recurrent acute calculous cholecystitis The white arrow indicates a gallbladder remnant that is distended with cholelithiasis, wall thickening, and pericholecystic fluid.

**Figure 2:**
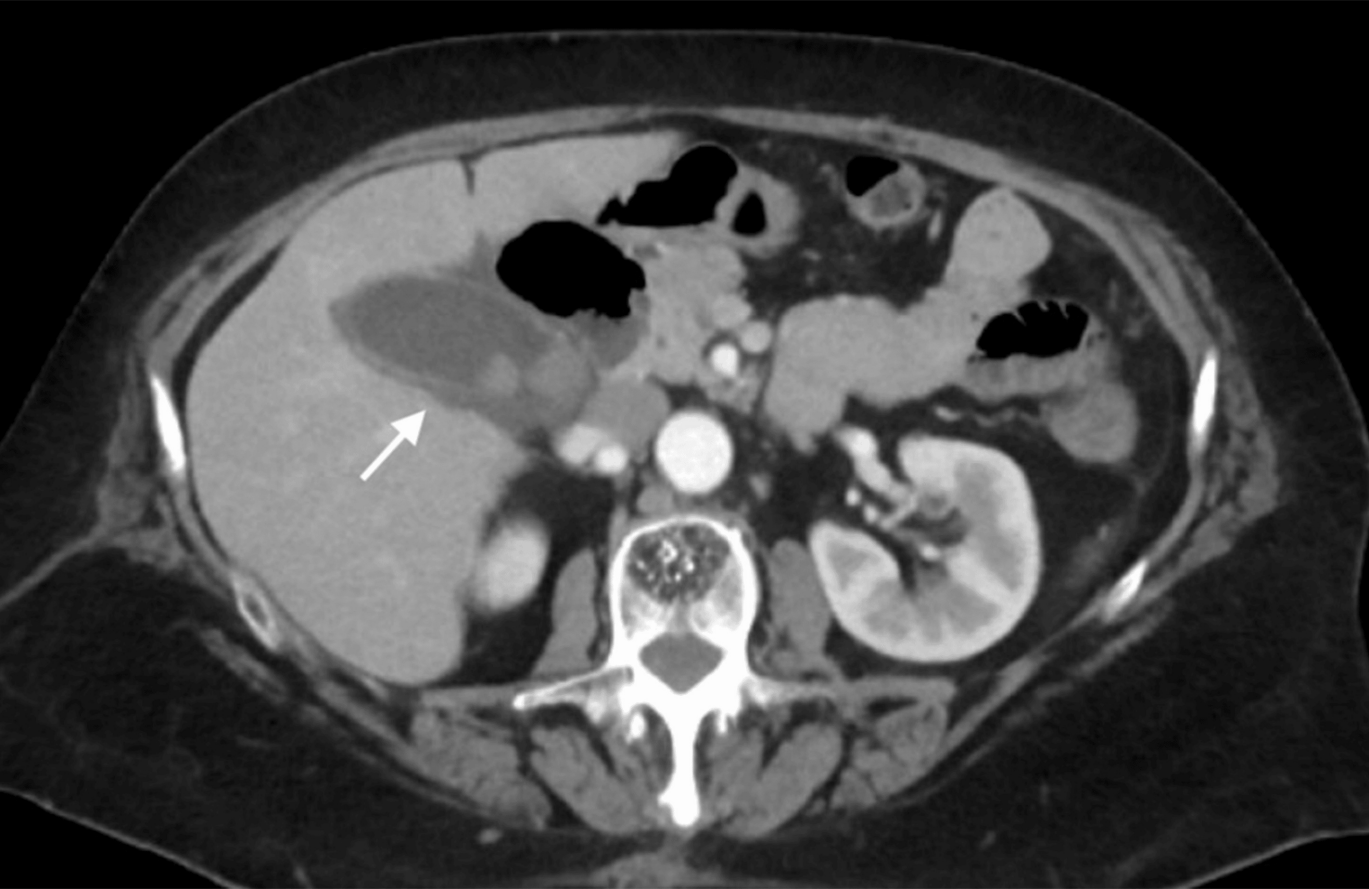
Axial CT image showing recurrent acute calculous cholecystitis The white arrow indicates a gallbladder remnant that is distended with cholelithiasis, wall thickening, and pericholecystic fluid.

A contrast-enhanced CT scan of the abdomen and pelvis was ordered instead of an ultrasound due to the patient’s history of prior cholecystectomy, which broadens the differential diagnosis beyond biliary pathology to include conditions such as pancreatitis, hepatic pathology, perforated viscus, or malignancy. Imaging revealed a distended gallbladder measuring 33 mm × 71 mm with cholelithiasis, wall thickening, and pericholecystic fluid, consistent with acute calculous cholecystitis. No evidence of choledocholithiasis was noted (Figure 1, Figure 2).

**Figure 3:**
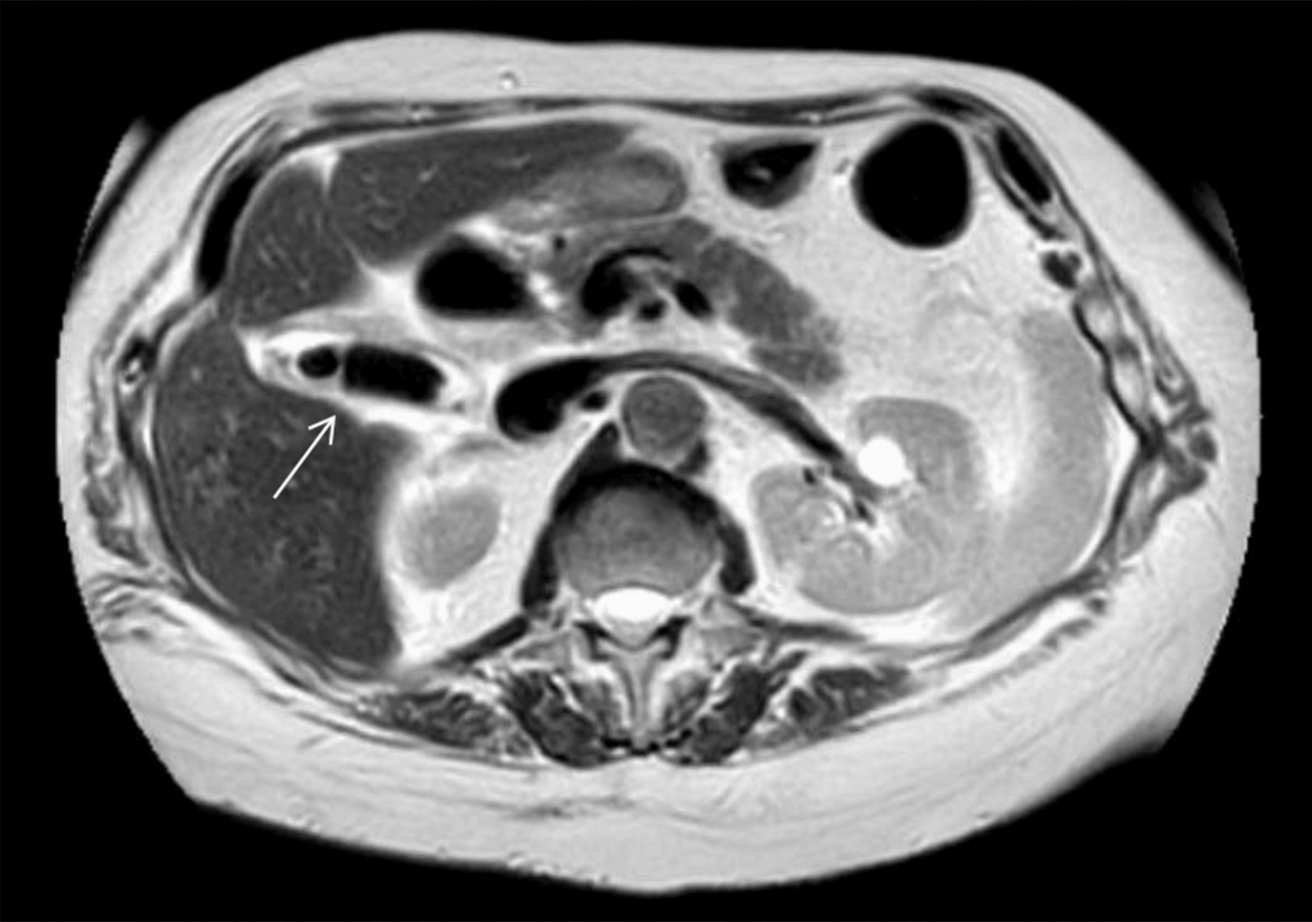
Axial MRCP image showing recurrent acute calculous cholecystitis The white arrow indicates a gallbladder remnant that is distended with cholelithiasis, wall thickening, and pericholecystic fluid. MRCP, magnetic resonance cholangiopancreatography

The patient was admitted under surgical consultation and started on intravenous antibiotics. An MRCP was obtained to assist with surgical planning and confirmed the diagnosis of acute cholecystitis (Figure 3).

The surgical team opted for placement of a percutaneous cholecystostomy tube due to the difficult anatomy visualized on MRCP, with plans for a subsequent complete cholecystectomy. The patient’s symptoms improved, she tolerated oral intake, and she was discharged home on a seven-day course of oral Augmentin with outpatient surgical follow-up.

In August 2025, a definitive laparoscopic cholecystectomy was performed. The procedure was complex, with extensive adhesions in the RUQ requiring over 90 minutes to take down; the omentum, transverse colon, and stomach were adherent to the gallbladder. The cystic duct was identified and clipped, and the gallbladder remnant was removed. The surgery lasted a total of five hours and 23 minutes, with an estimated blood loss of 60 mL. The postoperative course was uncomplicated: the patient’s pain improved, she tolerated diet, and she was discharged on postoperative day 1.

## Discussion

This case highlights important considerations in patients presenting with RUQ pain despite a history of laparoscopic cholecystectomy. The reported incidence of STC is approximately 0.52%, increasing from 0.10% prior to 2003. STCs reduce the rates of open total cholecystectomy when significant adhesions are present or when access to or visualization of critical biliary structures is compromised. From 2003 to 2014, the rate of open total cholecystectomy decreased from 10.5% to 7.6% [7].

In patients with prior cholecystectomy, especially when operative notes indicate a subtotal procedure or difficult dissection with residual gallbladder tissue, clinicians should maintain a differential that includes gallbladder remnant pathology. Approximately 10% of patients who undergo STC develop symptoms such as nausea, vomiting, and RUQ pain [8]. Although infrequent, retained gallbladder remnants may form cholelithiasis and subsequently develop acute cholecystitis. The rates of symptomatic gallstones and common bile duct stones have both been reported at 4% following STC, with 2% of cases requiring completion to total cholecystectomy [9].

Diagnostic imaging, including CT and MRCP, can reveal findings similar to, and often indistinguishable from, primary cholecystitis, including gallbladder distension, wall thickening, and pericholecystic fluid [3,6]. While no single imaging modality is considered the gold standard, multiple modalities are often used to facilitate diagnosis and surgical planning. Patients with recurrent cholecystitis require definitive management with complete removal of the gallbladder remnant. Subsequent surgical interventions are typically more complex due to fibrosis and adhesions from prior cholecystectomy [10]. Because of the challenging nature of these cases, placement of a percutaneous cholecystostomy tube prior to surgery, as in this case, is common [11].

This case underscores the importance of recognizing the risk of recurrent cholecystitis in patients with a history of gallbladder removal, particularly following STC.

## Conclusions

This case describes a rare instance of recurrent acute cholecystitis following STC. Subtotal cholecystectomies are performed to minimize injury to vital biliary structures and have reduced the rate of conversion to open cholecystectomy. Although STCs decrease perioperative complications, they are not without postoperative risks. This case emphasizes that in patients with a history of cholecystectomy, particularly following STC, who present with RUQ pain and signs of acute cholecystitis, clinicians should consider gallbladder remnant cholecystitis in the differential diagnosis. CT and MRCP imaging are critical for confirming the diagnosis, with definitive management requiring total cholecystectomy.

## References

[1] Elshaer M, Gravante G, Thomas K, Sorge R, Al-Hamali S, Ebdewi H (2015). Subtotal cholecystectomy for "difficult gallbladders": systematic review and meta-analysis. JAMA Surg.

[2] Walsh RM, Ponsky JL, Dumot J (2002). Retained gallbladder/cystic duct remnant calculi as a cause of postcholecystectomy pain. Surg Endosc.

[3] Demetriades H, Pramateftakis MG, Kanellos I, Angelopoulos S, Mantzoros I, Betsis D (2008). Retained gallbladder remnant after laparoscopic cholecystectomy. J Laparoendosc Adv Surg Tech A.

[4] Strasberg SM, Pucci MJ, Brunt LM, Deziel DJ (2016). Subtotal cholecystectomy-"fenestrating" vs "reconstituting" subtypes and the prevention of bile duct injury: definition of the optimal procedure in difficult operative conditions. J Am Coll Surg.

[5] Chowbey P, Sharma A, Goswami A (2015). Residual gallbladder stones after cholecystectomy: a literature review. J Minim Access Surg.

[6] Terhaar OA, Abbas S, Thornton FJ (2005). Imaging patients with "post-cholecystectomy syndrome": an algorithmic approach. Clin Radiol.

[7] Sabour AF, Matsushima K, Love BE, Alicuben ET, Schellenberg MA, Inaba K, Demetriades D (2020). Nationwide trends in the use of subtotal cholecystectomy for acute cholecystitis. Surgery.

[8] Shin M, Choi N, Yoo Y, Kim Y, Kim S, Mun S (2016). Clinical outcomes of subtotal cholecystectomy performed for difficult cholecystectomy. Ann Surg Treat Res.

[9] Al-Azzawi M, Abouelazayem M, Parmar C (2024). A systematic review on laparoscopic subtotal cholecystectomy for difficult gallbladders: a lifesaving bailout or an incomplete operation?. Ann R Coll Surg Engl.

[10] Jebakumar GS, Muthiah J, Jayapal LN (2024). Laparoscopic management of remnant gall bladder with stones: lessons from a tertiary care centre's experience. Laparosc Endosc Robot Surg.

[11] Teshima T, Nitta H, Mitsuura C (2021). How to treat remnant cholecystitis after subtotal cholecystectomy: two case reports. Surg Case Rep.

